# Mobility of primary health care workers in China

**DOI:** 10.1186/1478-4491-7-24

**Published:** 2009-03-17

**Authors:** Qingyue Meng, Jing Yuan, Limei Jing, Junhua Zhang

**Affiliations:** 1 Center for Health Management and Policy, Shandong University, Jinan, Shandong, PR China; 2 Health Human Resources Development Center, Ministry of Health, Beijing, PR China

## Abstract

**Background:**

Rural township health centres and urban community health centres play a crucial role in the delivery of primary health care in China. Over the past two-and-a-half decades, these health institutions have not been as well developed as high-level hospitals. The limited availability and low qualifications of human resources in health are among the main challenges facing lower-level health facilities. This paper aims to analyse the mobility of health workers in township and community health centres.

**Methods:**

Data used in this paper come from a nationwide survey of health facilities in 2006. Ten provinces in different locations and of varying levels of economic development were selected. From these provinces, 119 rural township health centres and 89 urban community health centres were selected to participate in a questionnaire survey. Thirty key informants were selected from these health facilities to be interviewed.

**Results:**

In 2005, 8.1% and 8.9% of health workers left township and community health centres, respectively. The health workers in rural township health centres had three to 13 years of work experience and typically had received a formal medical education. The majority of the mobile health workers moved to higher-level health facilities; very few moved to other rural township health centres. The rates of workers leaving township and community health centres increased between 2000 and 2005, with the main reasons for leaving being low salaries, limited opportunities for professional development and poor living conditions.

**Conclusion:**

In China, primary health workers in township health centres and community health centres move to higher-level facilities due to low salaries, limited opportunities for promotion and poor living conditions. The government already has policies in place to counteract this migration, but it must step up enforcement if rural township health centres and urban community centres are to retain health professionals and recruit qualified health workers.

## Background

China's health care system features a three-tiered system of health providers. In rural areas, village clinics, township health centres (THCs) and county hospitals are the major health care providers. In urban areas, community health centres (CHCs), district hospitals and municipal and provincial hospitals are the major providers. In this system, THCs and CHCs link the lower and upper levels of health providers and take a lead role in providing both curative and preventive care to local communities. In 2007, China had nearly 40 000 rural THCs with 860 000 health workers and 3200 urban CHCs with 106 100 health workers [1].

The operation of rural THCs has been challenged over the past two-and-a-half decades since economic reform began in China. Compared with upper-level health providers, THCs are at a disadvantage in terms of mobilizing resources for their development in a market-oriented health care system. Upper-level hospitals absorb the majority of qualified health professionals and high technologies. People with high incomes prefer to seek care from upper-level health providers [2].

Because user fees are the major source of financing for THCs, a decrease in health care utilization results in financial difficulties. In 2006, only 6.5% of total health expenditure was allocated to THCs, while health workers in THCs accounted for 20% of all health workers in China [3]. Decreased health care utilization and financial troubles weakened the ability of THCs to recruit and retain qualified health workers [4]. The urban community health care system was rebuilt at the end of the 1990s. Even though the number of CHCs has increased rapidly over the past decade, the quality of health care provided by CHCs is a concern [5]. A lack of both qualified health workers and government financial support are among the main reasons behind this low quality of care.

China's central government has clarified its goal of establishing a universal health care system [6]. One of the strategies for achieving this goal is to strengthen the primary health care system, focusing especially on THCs and CHCs. Human resources in THCs and CHCs would be the key factor in determining their performance. There have been few studies on the mobilization of primary health care workers in China, with the exception of a few papers looking at mobilization in a single hospital [7,8]. The purpose of this paper is to analyse the mobilization of primary health workers in China using nationwide survey data.

## Methods

The data used in this study come from a nationwide facility-based survey conducted in 2006. Ten provinces were selected according to their location and level of economic development. In each of the provinces, six rural counties and four cities were selected. In each of the counties, two THCs were selected, while in each of the cities, three CHCs were selected. A total of 119 rural THCs and 89 urban CHCs were selected for a questionnaire survey. From these facilities, 30 heads of the THCs and CHCs were selected for key-informant interviews.

Investigators came from Shandong University and the Health Human Resources development Center, Ministry of Health. Indicators in the facility-based questionnaire included total number of health workers, number of health workers who had left the facility, characteristics of health workers who left the facility (length of work experience and educational background) and the institution to which the health workers moved. The questionnaire was completed by personnel officers at the selected THCs and CHCs with instructions from the investigators.

Question guidelines were used in the key-informant interviews. Questions for the review included why some health professionals would leave for new institutions and how health professionals can be retained by THCs and CHCs. The interviews were conducted by the investigators in the interviewee's workplace.

## Results

### Number, experience and education of primary health workers who move

In 2005, an average of two health workers per THC and 2.2 health workers per CHC left their working institutions, excluding retirements. In the same year, average total health workers numbered 24.7 and 24.6 in THCs and CHCs, respectively. Health workers who moved to other institutions accounted for 8.1% and 8.9% of the total health workers in THCs and CHCs.

The health workers who left THCs for other institutions had work experience ranging from three to 13 years. The work experience of health workers who left CHCs was somewhat shorter, ranging from one to six years.

In THCs, 29% of total health workers had received a three- to five-year formal medical education in colleges or universities. The majority of health workers had not received any higher-level medical education. Of the two health workers who left the THCs in 2005, one had received formal medical education. In CHCs, 73.2% of health workers had received formal medical education. Of the 2.2 health workers who left each CHC, an average of 1.3 had received high medical education.

### Proportion of approaches for leaving

There are two ways in which a health worker can move from his or her current institution to other institutions. The "normal" way is that the move is agreed upon by the current institution. The other is resignation, implying that the institution does not agree but the health worker insists on leaving regardless. Health institutions usually rely on the latter approach in an attempt to retain the workers. Table 1 shows the proportions of health workers who left their institutions between 2001 and 2005. Resignations increased by 6.4% and 10% in THCs and CHCs, respectively, from 2001 to 2005.

**Table 1:** Outflow of health workers from THCs and CHCs (%)

Year	**THCs**			**CHCs**		
	
	Normal flow	Resignation	Other reasons	Normal flow	Resignation	Other reasons
2001	73.3	11.1	15.6	71.4	21.4	7.2

2002	65.6	18.3	16.1	68.4	7.9	23.7

2003	62.8	15.4	21.8	63.6	18.2	18.2

2004	70.5	15.5	14.0	56.5	34.8	8.7

2005	65.6	17.5	16.9	51.5	31.3	17.2

### Distribution of health workers who moved

More than 50% of the health workers went to higher-level health institutions after they left the THCs and CHCs (Table 2 and Table 3). These higher-level health facilities were usually county hospitals in rural areas and district or municipal hospitals in urban areas. The proportion of health workers moving to higher-level health facilities decreased from 2001 to 2005, with high proportions of health workers leaving CHCs for non-health facilities in 2001 and 2002. A very small proportion of health workers left for another health facility of the same level as the one they left.

**Table 2:** Distribution of primary health workers who moved from THCs (%)

**Year**	**Higher-level health facilities**	**Non-health institutions**	**Same-level health facilities**	**Others**
2001	75.0	5.0	0	20.0

2002	60.2	10.3	2.6	26.9

2003	56.0	5.3	5.3	33.4

2004	62.9	8.1	2.4	26.6

2005	54.9	6.3	4.9	33.9

**Table 3:** Distribution of primary health workers who moved from CHCs (%)

**Year**	**Higher-level health facilities**	**Non-health institutions**	**Same-level health facilities**	**Others**
2001	69.2	30.8	0	0

2002	60.2	27.0	8.1	4.7

2003	56.8	6.8	2.3	34.1

2004	54.5	4.5	6.1	34.9

2005	57.3	6.1	1.2	35.4

### Reasons for leaving THCs and CHCs

Salary, opportunities for professional development and living conditions were the most frequently cited reasons for moving. The following are key messages from interviewees.

"Income is lower in THCs than in higher level hospitals. Higher level hospitals can offer bonuses for their health workers besides salaries. We cannot, because our ability to generate revenues is limited. As head of this THC, my concern is that some qualified health professionals within the THC may want to leave because of low income. One good and experienced physician left our THC last year mainly due to income" (the head of a THC in Zongyang County of Anhui Province).

"Health workers, especially new graduates from medical universities, feel that there are limited opportunities for medical practice here than at higher-level hospitals. In addition, there is a lack of adequate financing to support health workers to attend training programmes outside the THCs. Some health workers try to leave for higher-level health facilities that have more opportunities for their professional development" (the head of a THC in Rongshui County of Guangxi Province).

"It is hard for us to recruit graduates of medical universities. The main reason for this is that they realize that there are fewer opportunities in CHCs than in upper-level health facilities for professional promotion and development" (the head of a CHC from Hangzhou Municipal City of Zhejiang Province).

"Some health workers, especially young ones, do not like to stay in THCs, even if the income of health workers in THCs is a little bit higher than that in higher-level hospitals. This is because the living conditions, including children's education, in the rural town where the THCs are located are generally poorer than in the county town" (the head of a THC in Lanxi County of Zhejiang Province).

## Discussion

A high proportion of THC and CHC health workers moved to high-level health facilities or non-health institutions. The THCs and CHCs find it difficult to control this emigration because health workers are free to resign from their current workplace. Low salaries, limited opportunities for professional development and unsatisfactory living conditions were the main reasons why the health workers left.

From the mid-1980s to the present, China's health sector has been expanding rapidly. The number of health professionals increased from 4.5 million in 2000 to 4.8 million in 2007 [1]. However, the number of health professionals in THCs decreased from 1 million to 0.86 million over the same time period [1].

At the same time, it is interesting to note that between 2000 and 2007, the average number of health professionals in each THC and CHC increased slightly, by 0.9 and 0.7, respectively [1]. For THCs, this is largely due to a reduction in the number of THCs as townships were combined and reorganized in the early 2000s.

Compared with the mobility rates of health workers in THCs and CHCs, hospitals at and above the county level had much lower proportions (only 2.5%) of health workers moving to other institutions [9]. Neither the quantity nor quality of health professionals in THCs met the health needs of local communities, especially in poor rural counties [10]. A high proportion of health workers leaving THCs and CHCs would have a significant impact on provision of health care. Furthermore, health workers leaving THCs and CHCs are usually experienced and qualified health professionals, possibly because these workers can more easily find new positions in high-level health facilities.

Since the early 1980s, the relationship between different levels of health providers has changed from partnership to competition, because health providers rely on user fees to generate revenues for their operation. High-level health providers, including county hospitals in rural counties and municipal hospitals in urban areas, are better positioned to compete for resources. Gaps in both income and opportunities for professional development have widened between low- and high-level health facilities. As a result, high-level facilities are able to recruit qualified health professionals away from THCs and CHCs.

The personnel policy for health worker mobility has been adjusted by the government. Before the mid-1980s, mobility of employees between public institutions was highly restricted by the government. Workers who wished to move to other institutions had to have their applications approved by their original working institution. This policy has since been changed [11]. Now, public sector employees are free to leave their current institution for another institution as long as the new institution agrees. This more flexible policy, combined with better pay at high-level facilities, has led to an increasing number of health professionals leaving THCs and CHCs.

In recent years, the government has tried to encourage health professionals to work in primary health facilities and to train medical graduates for those facilities [12,13]. Key policy strategies include increasing salaries for health professionals working in primary health facilities, creating more promotion opportunities for primary health professionals and offering more training opportunities.

While these policies effectively target primary health workers' concerns about income and opportunities for professional development, they have not been well-enforced in practice. A survey in Qinghai Province indicated that problems of low income and limited opportunity of professional development of health workers have not been addressed [14]. In poor areas, few training opportunities have been created for health workers in THCs [15]. While the capacity of higher medical education has been greatly expanded – total enrolments in medical universities increased from 0.42 million in 2000 to 1.13 million in 2005 [16] – few graduates want to work in primary health facilities. Some graduates from medical universities would rather take a non-medical job in an urban area than work at a low-level health facility in a rural area [17].

## Conclusion

In China, primary health workers in THCs and CHCs move to high-level health facilities due to relatively low salaries, limited opportunities for promotion and poor living conditions. The government already has policies in place to counteract this migration, but it must step up enforcement if THCs and CHCs are to retain health professionals and recruit qualified health workers.

## Competing interests

The authors declare that they have no competing interests.

## Authors' contributions

MQ contributed to design, methods, fieldwork and writing. YJ contributed to methods, fieldwork and report writing. JL contributed to fieldwork and ZJ contributed to design and fieldwork.
